# Serum levels of immunoglobulins, tumour markers, and chemokines in gastric cancer patients treated with SOX regimen versus oxaliplatin monotherapy

**DOI:** 10.5937/jomb0-56172

**Published:** 2026-05-22

**Authors:** Ying Huang, Yueming Hu, Fengwei Gu, Yufeng Ni

**Affiliations:** 1 Chongming Hospital, Affiliated to Shanghai University of Medicine & Health Sciences, Department of Oncology, Shanghai, China; 2 Chongming Hospital, Affiliated to Shanghai University of Medicine & Health Sciences, Department of Gastroenterology, Shanghai, China

**Keywords:** gastric cancer, biomarkers, SOX regimen, immunoglobulins, tumour markers, chemotherapy response, karcinom želuca, biomarkeri, SOX režim, imunoglobulini, tumorski markeri, odgovor na hemoterapiju

## Abstract

**Background:**

Gastric cancer is a leading cause of cancer-related mortality, emphasising the need for reliable bio-markers for prognosis and treatment monitoring. This study evaluates serum levels of immunoglobulins (IgA, IgG, IgM), cancer markers (CA125, CEA), and other biomarkers (MRP-14, SDF-1, FSP-1, CXCR4) in gastric cancer patients after treatment.

**Methods:**

100 gastric cancer patients were randomised into two treatment groups: oxaliplatin plus capecitabine (reference) and the SOX regimen (oxaliplatin plus S-1; observation). Serum biomarker levels were measured before and after treatment using flow cytometry and enzyme immunoassays. Clinical efficacy, immune response, and chemotherapy-related toxicity were analysed.

**Results:**

The SOX regimen demonstrated superior clinical efficacy, with higher objective response (76% vs. 62%) and disease control rates (94% vs. 86%) compared to oxaliplatin monotherapy. The SOX group exhibited significantly higher post-treatment levels of IgA, IgG, and IgM and increased CD 3+, CD 4+, and NK cell counts. Tumour marker levels (CA125, CEA, MRP-14, SDF-1, FSP-1, CXCR4) were lower in the SOX group, with fewer chemotherapy-related adverse effects.

**Conclusions:**

The SOX regimen enhances immune function, reduces tumour markers, and improves treatment outcomes compared to oxaliplatin monotherapy. Serum bio-markers may be valuable for monitoring therapeutic responses in gastric cancer patients. However, the study's small sample size and retrospective design limit the generalizability of the findings. Further studies are necessary to confirm these results and validate the prognostic value of serum biomarkers.

## Abstract (sr)

**Uvod:**

Karcinom želuca je jedan od vodećih uzroka mortaliteta povezanog sa rakom, što naglašava potrebu za pouzdanim biomarkerima za prognozu i praćenje terapije. Ova studija procenjuje serumske nivoe imunoglobulina (IgA, IgG, IgM), tumorskih markera (CA125, CEA) i drugih biomarkera (MRP-14, SDF-1, FSP-1, CXCR4) kod pacijenata sa karcinomom želuca nakon terapije.

**Metode:**

Sto pacijenata sa karcinomom želuca je nasumično podeljeno u dve grupe terapije: oksaliplatin plus kapecitabin (referentna grupa) i SOX režim (oksaliplatin plus S-1; posmatrana grupa). Nivoi serumskih biomarkera mereni su pre i nakon terapije primenom protočne citometrije i enzimskih imunoeseja. Analizirani su klinička efikasnost, imunološki odgovor i toksičnost povezana sa hemoterapijom.

**Rezultati:**

SOX režim je pokazao superiornu kliničku efikasnost, sa visokom stopom objektivnog odgovora (76% naspram 62%) i stopom kontrole bolesti (94% naspram 86%) u poređenju sa monoterapijom oksaliplatinom. Grupa lečena SOX režimom imala je značajno više postterapijske nivoe IgA, IgG i IgM, kao i povećan broj CD 3+, CD4+ i NK ćelija. Nivoi tumorskih markera (CA125, CEA, MRP-14, SDF-1, FSP-1, CXCR4) bili su niži u SOX grupi, uz manje neželjenih efekata povezanih sa hemoterapijom.

**Zaključak:**

SOX režim poboljšava imunološku funkciju, smanjuje nivo tumorskih markera i dovodi do boljih terapijskih ishoda u poređenju sa monoterapijom oksaliplatinom. Serumski biomarkeri mogu biti korisni za praćenje terapijskog odgovora kod pacijenata sa karcinomom želuca. Međutim, Mali uzorak i retrospektivni dizajn studije ograničavaju generalizaciju rezultata. Potrebne su dodatne studije radi potvrde nalaza i validacije prognostičke vrednosti serumskih biomarkera.

## Introduction

Gastric cancer is one of the most prevalent and deadly malignancies worldwide, ranking among the top five cancers in terms of incidence and mortality [1][2]. In 2020, the Global Cancer Statistics (GLOBOCAN) reported over one million new cases and approximately 770,000 deaths from gastric cancer, with the highest burden observed in East Asia, particularly China, Japan, and Korea [3]. Despite a decline in incidence in some regions due to improved hygiene, Helicobacter pylori eradication, and dietary modifications, gastric cancer remains a major global health concern [2][4]. The disease is often diagnosed at an advanced stage, as early-phase symptoms are usually asymptomatic, contributing to poor survival outcomes. Gastric cancer primarily arises from the gastric mucosa, with adenocarcinoma being the most common histological type [5].

The pathogenesis of gastric cancer is complex, driven by a combination of genetic predisposition, environmental factors, and chronic inflammation. Helicobacter pylori infection is a major etiological factor, leading to chronic gastritis, atrophic gastritis, intestinal metaplasia, and eventually malignant transformation [6][7]. Other risk factors include smoking, high-salt diets, alcohol consumption, obesity, and Epstein-Barr virus infection [8]. Molecular mechanisms underlying gastric cancer progression involve several key signalling pathways, such as Wnt/ - catenin, PI3K/Akt/mTOR, and epithelial-mesenchymal transition, which contribute to tumour proliferation, invasion, and metastasis [9][10].

Current management of gastric cancer depends on the disease stage, histology, and patient performance status. Early-stage cancer may be treated with endoscopic resection or radical gastrectomy with lymphadenectomy [11]. Locally advanced cases often benefit from perioperative chemotherapy or chemora-diotherapy to improve resectability and survival outcomes [12]. In metastatic gastric cancer, systemic chemotherapy is the primary treatment modality, with fluoropyrimidine-based regimens like capecitabine, 5-fluorouracil, oxaliplatin, and S-1 commonly used [13][14]. Targeted therapies, such as trastuzumab for HER2-positive gastric cancer and ramucirumab for anti-angiogenic treatment, have shown survival benefits. Additionally, immune checkpoint inhibitors like pembrolizumab and nivolumab are emerging as promising therapies for patients with microsatellite instability-high or PD-L1-expressing tumours [15]. Despite these advancements, treatment resistance and recurrence remain significant challenges, highlighting the need for reliable biomarkers to assess prognosis and monitor therapeutic response.

Biomarkers are essential in diagnosing, prognosticating, and evaluating treatment responses in gastric cancer. This study focuses on a specific set of biomarkers, including immunoglobulin A (IgA), immunoglobulin G (IgG), immunoglobulin M (IgM), cancer antigen 125 (CA125), carcinoembryonic antigen (CEA), migration inhibitory factor-related protein 14 (MIF-14), stromal cell-derived factor 1 (SDF-1), fibroblast-specific protein 1 (FSP1), and C-X-C motif chemokine receptor 4 (CXCR4), in gastric cancer patients after treatment. These biomarkers were selected based on their potential to reflect various aspects of gastric cancer progression and treatment response.

Immunoglobulins (IgA, IgG, IgM) serve as indicators of host immune function and response to cancer, with changes in their levels possibly reflecting tumour burden, immune suppression, or immune modulation due to treatment. CA125 and CEA have established tumour markers commonly associated with gastrointestinal malignancies. Elevated levels of these markers are linked to tumour progression, metastasis, and poor prognosis, making them valuable for assessing treatment response [16][17]. MIF-14 is a pro-inflammatory protein involved in cancer-related inflammation and immune evasion; increased levels of MIF-14 are associated with poor clinical outcomes in several cancers, including gastric cancer [18][19][20]. SDF-1 plays a critical role in tumour-stroma interactions, angiogenesis, and metastasis by recruiting circulating tumour cells and supporting tumour cell survival in distant organs [21]. FSP1 is a marker of cancer-associated fibroblasts, which contribute to tumour progression and chemoresistance, with elevated levels indicating a more aggressive tumour microenvironment [22]. CXCR4 is a key regulator of tumour cell migration and metastasis; its overexpression in gastric cancer is linked to enhanced metastatic potential and poor prognosis [23].

This study hypothesises that these biomarkers can serve as valuable indicators for monitoring treatment response, immune status, and their potential correlation with clinical outcomes in gastric cancer patients. By analysing these markers before and after treatment, the study aims to enhance the understanding of their role in predicting prognosis and facilitating more personalised treatment strategies.

## Materials and methods

### Study participants

Following the exclusion of eight ineligible cases, 100 patients diagnosed with gastric cancer were enrolled in this study. Participants were randomly assigned, in a 1:1 ratio, to receive either oxaliplatin (reference group) or the SOX regimen (observation group) using a random number table method, with 50 patients allocated to each group. The ethics committee of our hospital approved the study protocol, and all patients provided written informed consent prior to participation.

### Inclusion criteria

Patients were eligible for inclusion if they met the following criteria:

1. Diagnosed with advanced gastric cancer and exhibiting normal results in electrocardiography (ECG), routine blood tests, and liver and kidney function tests.
2. Aged 18 years or older, irrespective of gender.
3. Expected survival of at least six months.
4. A Karnofsky Performance Status (KPS) score above 60.

### Exclusion criteria

Patients were excluded from the study if they met any of the following conditions:

1. A known allergy or intolerance to the study medications.
2. A history of organ transplantation.
3. Pregnancy or lactation.
4. Presence of brain metastases or impaired consciousness.
5. Diagnosis of pulmonary fibrosis.

### Treatment protocol

### Drug sources

The pharmaceutical agents used in this study were sourced from the following manufacturers:

1. Oxaliplatin (Approval No. H20133247) - Shandong New Age Pharmaceutical Co.
2. Capecitabine - Shanghai Roche Pharmaceutical Co.
3. Tegafur/gimeracil/oteracil (S-1) (Approval No. H20080802) - Lunan Pharmaceutical Group Co.

### Reference group (Oxaliplatin + Capecitabine)

Patients in the reference group received premedication with diphenhydramine and cimetidine 30 minutes before drug administration to prevent allergic reactions. Oxaliplatin was administered at a dose of 130 mg/m^2^ via intravenous infusion over more than two hours on Day 1. This was followed by oral capecitabine at a dosage of 1000 mg/m^2^, taken twice daily from Days 1 to 14. The treatment regimen consisted of two cycles, with each cycle lasting 21 days.

### Observation group (SOX regimen)

Patients in the observation group received oxaliplatin at a dose of 130 mg/m^2^ via intravenous infusion over more than two hours on Day 1, followed by oral administration of S-1 (40 mg) twice daily from Days 1 to 14. The treatment regimen comprised two cycles of 21 days each. During drug administration, patients were advised to avoid exposure to cold and direct contact with cold objects.

### Clinical endpoints

1. Tumour Response Assessment: Following treatment, tumour size (total lesion length and width) was evaluated using magnetic resonance imaging (MRI) and gastroscopy. Tumour response was classified according to the following criteria:<br>Complete response (CR): Total disappearance of lesions persisting for more than 28 days.<br>Partial response (PR): A reduction of 30% in total lesion length and width persisting for more than 28 days.<br>Stable disease (SD): A lesion reduction that does not meet the criteria for PR and has a duration of less than 28 days.<br>Progressive disease (PD): A reduction smaller than that defined for SD.<br>Objective response rate (ORR): (CR + PR) / total number of cases × 100%.<br>Disease control rate (DCR): (CR + PR + SD) / total number of cases × 100%.
2. Immunological and Biomarker Assessment: Before and after treatment, 5 mL of fasting venous blood was collected from each patient. Serum levels of immunoglobulin A (IgA), immunoglobulin G (IgG), and immunoglobulin M (IgM) were measured via flow cytometry. Additionally, T lymphocyte and natural killer (NK) cell ratios, as well as tumour biomarkers associated with gastric cancer, were assessed using enzyme immunoassay. The biomarkers analysed included cancer antigen 125 (CA125), carcinoembryonic antigen (CEA), migration inhibitory factor-related protein 14 (MRP14), stromal cell-derived factor 1 (SDF-1), fibroblast-specific protein 1 (FSP-1), and C-X-C Motif Chemokine Receptor 4 (CXCR4).
3. Chemotherapy Toxicity Assessment: Chemotherapy-related toxicities were evaluated based on the National Cancer Institute Common Toxicity Criteria (NCI-CTC).

### Statistical analysis

Statistical analyses were conducted using SPSS 26.0 software. Continuous variables were expressed as mean±standard deviation (x̄±s) and compared using the independent samples t-test. Categorical variables were presented as percentages (%) and analysed using the chi-square (χ^2^) test. A p-value of <0.05 was considered statistically significant.

## Results

### Patient characteristics and demographics

The observation group comprised 28 male and 22 female patients, with an age range of 44 to 73 years (mean: 60.58±7.35 years). All patients in this group had a Karnofsky Performance Status (KPS) score greater than 60 (mean: 76.98±5.23). Regarding tumour classification, 19 patients were diagnosed with TNM stage IIIb, while 31 patients had stage IV disease. Histopathological analysis revealed 30 cases of adenocarcinoma, 5 cases of mucinous adenocarcinoma, and 15 cases of indolent cell carcinoma.

The reference group included 26 male and 24 female patients, aged between 47 and 78 years (mean: 60.88±7.17 years), all with a KPS score above 60 (mean: 76.24±5.05). Within this cohort, 21 patients were classified as TNM stage IIIb, while 29 were diagnosed with stage IV disease. The histopathological distribution consisted of 33 cases of adenocarcinoma, 6 cases of mucinous adenocarcinoma, and 11 cases of indolent cell carcinoma.

A statistical analysis demonstrated no significant differences between the two groups concerning demographic and clinical characteristics (P>0.05), indicating comparability between cohorts (Table 1).

**Table 1:** Patient characteristics (x̄ ± s).

		Observation group	Reference group	*t*	*P*
n	-	50	50	-	-
Sex	Male	28 (56.00)	26 (52.00)	-	-
	Female	22 (44.00)	24 (48.00)	-	-
Age (years)	-	44-73	47-78	-	-
	Mean	60.58±7.35	60.88±7.17	0.207	0.837
KPS scores	-	>60	>60	-	-
	Mean	76.98±5.23	76.24±5.05	0.719	0.474
TNM stage	IIIb	19 (38.00)	21 (42.00)	-	-
	IV			-	-
Pathological type	Adenocarcinoma	30 (60.00)	33 (66.00)	-	-
	Mucinous adenocarcinoma	5 (10.00)	6 (12.00)	-	-
	Indocellular carcinoma	15 (30.00)	11 (22.00)	-	-

### Clinical efficacy

In the observation group, 20 patients achieved complete response (CR), 18 exhibited partial response (PR), nine experienced stable disease (SD), and 3 demonstrated progressive disease (PD). The disease control rate (DCR) in this group was 94.00%, while the objective response rate (ORR) was 76.00%.

**Figure 1:**
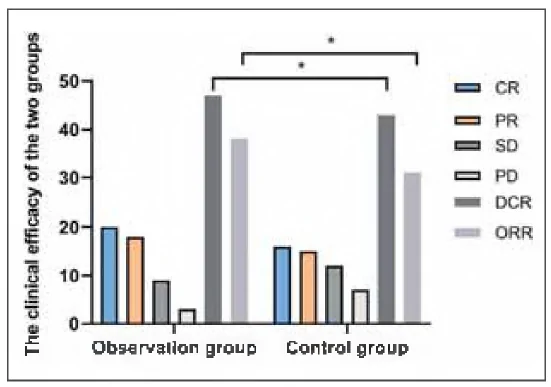
Clinical efficacy. Note: * indicates P < 0.05

In the reference group, 16 patients achieved CR, 15 had PR, 12 maintained SD, and 7 showed PD. The corresponding DCR was 86.00%, and the ORR was 62.00%. Statistical analysis revealed that the SOX regimen significantly improved clinical efficacy compared to oxaliplatin monotherapy, as evidenced by superior DCR and ORR (P<0.05) (Figure 1).

### Immunoglobulin levels

Following treatment, the observation group demonstrated elevated immunoglobulin levels, with IgA at 2.07±0.58 g/L, IgG at 7.56±2.01 g/L, and IgM at 0.53±0.13 g/L. In contrast, the reference group exhibited lower post-treatment immunoglobulin levels, with IgA at 1.53±0.44 g/L, IgG at 6.03±1.88 g/L, and IgM at 0.45±0.18 g/L. These findings suggest that the SOX regimen facilitates enhanced immune recovery relative to oxaliplatin monotherapy, as indicated by significantly higher IgA, IgG, and IgM levels (P<0.05) (Table 2).

**Table 2:** Immunoglobulins (x̄ ± s).

		Observation group	Reference group	*t*	*P*
n	-	50	50	-	-
Before treatment	IgA (g/L)	1.16±0.33	1.18±0.34	0.298	0.766
	IgG (g/L)	5.11±1.39	5.09±1.48	0.070	0.945
	IgM (g/L)	0.39±0.08	0.38±0.11	0.022	0.983
After treatment	IgA (g/L)	2.07±0.58	1.53±0.44	5.345	<0.001
	IgG (g/L)	7.56±2.01	6.03±1.88	3.931	<0.001
	IgM (g/L)	0.53±0.13	0.45±0.18	2.628	0.010

### T-cell subsets and NK cells

In the observation group, post-treatment levels of CD3+, CD4+, CD8+, and natural killer (NK) cells were 61.99±4.52, 39.54±2.28, 28.14±2.21, and 8.46±0.56, respectively. Corresponding values in the reference group were 58.41±4.33 for CD3+, 36.25±2.37 for CD4+, 35.01±2.66 for CD8+, and 6.15±0.81 for NK cells. These results indicate that the SOX regimen was more effective in enhancing serum levels of T-cell subsets and NK cells than oxaliplatin alone (P<0.05) (Table 3).

**Table 3:** T-cell subsets and NK cells (x̄ ± s).

		Observation group	Reference group	*t*	*P*
n	-	50	50	-	-
Before treatment	CD3+	57.15±3.68	57.34±3.55	0.249	0.804
	CD4+	46.14±3.08	46.22±3.35	0.125	0.901
	CD8+	37.85±2.78	37.74±2.94	0.193	0.848
	NK cells	9.41±1.01	9.52±1.03	0.542	0.589
After treatment	CD3+	61.99±4.52	58.41±4.33	4.042	<0.001
	CD4+	39.54±2.28	36.25±2.37	7.088	<0.001
	CD8+	28.14±2.21	35.01±2.66	14.095	<0.001
	NK cells	8.46±0.56	6.15±0.81	16.696	<0.001

### Tumour markers

Serum levels of tumour markers were significantly reduced in the observation group compared to the reference group. Specifically, the observation group had CA125 levels of 48.89±7.65 U/mL, CEA levels of 40.56±8.41 ng/mL, MRP-14 levels of 8.14±1.18 ng/mL, SDF-1 levels of 2.64±0.96 ng/mL, FSP-1 levels of 4.35±1.14 ng/mL, and CXCR4 levels of 0.48±0.08 ng/mL.

Conversely, the reference group exhibited markedly higher tumour marker levels, with CA125 at 101.84±10.51 U/mL, CEA at 68.56±9.12 ng/mL, MRP-14 at 11.74±1.33 ng/mL, SDF-1 at 4.97±1.35 ng/mL, FSP-1 at 8.56±1.94 ng/mL, and CXCR4 at 0.82±0.13 ng/mL. These findings suggest that the SOX regimen provides significantly more significant anti-tumour benefits compared to oxaliplatin monotherapy, as indicated by the reduced serum concentrations of these tumour markers (P<0.05) (Table 4).

**Table 4:** Tumour markers (x̄ ± s).

		Observation group	Reference group	*t*	*P*
n	-	50	50	-	-
Before treatment	CA125 (U/mL)	247.56±19.55	247.88±19.68	0.082	0.935
	CEA (ng/mL)	125.26±14.35	124.98±13.97	0.099	0.922
	MRP-14 (mg/L)	30.56±4.15	30.88±4.03	0.384	0.702
	SDF-1 (ng/L)	9.56±2.08	9.47±2.05	0.242	0.809
	FSP-1 (ng/L)	18.56±2.41	18.94±2.17	0.829	0.409
	CXCR4 (pg/L)	1.53±0.21	1.55±0.19	0.762	0.448
After treatment	CA125 (U/mL)	48.89±7.65	101.84±10.51	28.788	<0.001
	CEA (ng/mL)	40.56±8.41	68.56±9.12	15.960	<0.001
	MRP-14 (mg/L)	8.14±1.18	11.74±1.33	14.263	<0.001
	SDF-1 (ng/L)	2.64±0.96	4.97±1.35	9.987	<0.001
	FSP-1 (ng/L)	4.35±1.14	8.56±1.94	13.230	<0.001
	CXCR4 (pg/L)	0.48±0.08	0.82±0.13	16.456	<0.001

### Toxicity and adverse effects

The SOX regimen demonstrated a superior safety profile compared to oxaliplatin monotherapy, as evidenced by a lower incidence of treatment-related adverse effects. Patients receiving the SOX regimen experienced significantly reduced rates of nausea, vomiting, and leukopenia (P<0.05) (Table 5).

**Table 5:** Toxic side effects (%).

	Observation group	Reference group	χ^2^	*P*
n	50	50	-	-
Anemia	19 (38.00)	20 (40.00)	0.042	0.838
Nausea and vomiting	6 (12.00)	14 (28.00)	4.000	0.046
Thrombocytopenia	10 (20.00)	17 (34.00)	2.486	0.115
Leukopenia	18 (35.00)	28 (56.00)	4.026	0.045
Mucositis of the oral cavity	5 (10.00)	3 (6.00)	0.543	0.461
Liver function impairment	4 (8.00)	4 (8.00)	0.000	1.000
Hand-foot syndrome	9 (18.00)	10 (20.00)	0.065	0.799
Peripheral neurotoxicity	8 (16.00)	7 (14.00)	0.078	0.779

## Discussion

This study showed that the SOX regimen (oxaliplatin plus S-1) significantly improved clinical efficacy compared to oxaliplatin monotherapy. The SOX regimen achieved a higher objective response (76% vs. 62%) and disease control rates (94% vs. 86%). Additionally, the SOX group exhibited enhanced immune function, as evidenced by significantly increased post-treatment levels of immunoglobulins (IgA, IgG, IgM) and T-cell subsets (CD3+, CD4+, NK cells). Tumour marker levels (CA125, CEA, MRp-14, SDF-1, FSP-1, CXCR4) were significantly reduced in the SOX group, suggesting improved tumour control and metastasis inhibition. Moreover, the SOX regimen was associated with fewer chemotherapy-related adverse effects, including reduced rates of nausea, vomiting, and leukopenia, highlighting its better tolerability.

Previous research has indicated that tumour markers are associated with chemotherapy response in various types of cancer [24][25][26]. However, their predictive significance in advanced gastric cancer remains uncertain [27][28]. The findings of this study demonstrate that the SOX regimen (oxaliplatin plus S-1) provides superior clinical efficacy in treating gastric cancer compared to oxaliplatin plus capecitabine. The SOX regimen yielded a significantly higher objective response rate (76.00% vs. 62.00%) and disease control rate (94.00% vs. 86.00%), indicating improved tumour response. These results align with previous studies that have reported enhanced treatment outcomes with S-1-based chemotherapy, likely due to its greater oral bioavailability and sustained cytotoxic effects compared to capecitabine.

One of the key findings of this study is the observed enhancement of immune function in the SOX group, as evidenced by increased levels of immunoglobulins (IgA, IgG, IgM) and T-cell subsets (CD3+, CD4+, NK cells). This suggests that the SOX regimen may exert immunomodulatory effects, potentially improving host anti-tumour immunity. The increase in immunoglobulin levels post-treatment may indicate enhanced B-cell activation, which plays a crucial role in immune surveillance against tumours. Moreover, the higher levels of CD3+ and CD4+ cells suggest improved T-cell-mediated immunity, which is essential for mounting an effective anticancer response.

Serum tumour marker levels were also significantly reduced in the SOX group, including CA125, CEA, MRP-14, SDF-1, FSP-1, and CXCR4. These biomarkers are associated with tumour progression, metastasis, and immune evasion in gastric cancer. The substantial decrease in their serum levels suggests that the SOX regimen effectively inhibits tumour growth and metastatic potential. Specifically, MRP-14 and SDF-1 reductions imply diminished inflammatory and stromal contributions to tumour progression, while decreased FSP-1 and CXCR4 levels indicate potential suppression of the tumour microenvironment's pro-metastatic activity.

Additionally, the SOX regimen was associated with fewer chemotherapy-related adverse effects compared to oxaliplatin plus capecitabine. The lower incidence of nausea, vomiting, and leukopenia suggests improved tolerability, which may enhance patient compliance and treatment adherence. This finding is particularly relevant as chemotherapy-related toxicity often limits treatment effectiveness and patient quality of life.

In a recent study by Du et al. [29], the Prognostic Nutritional Index (PNI) combined with immunoglobulin M (IgM) was proposed as a novel predictive marker for the prognosis of gastric cancer patients undergoing surgery. Their findings demonstrated that a lower PNI-IgM score correlated with poorer survival outcomes, highlighting the role of nutritional and immunological status in disease progression. Similarly, our study explores prognostic factors in gastric cancer, emphasising the impact of various clinical indicators on patient outcomes. By integrating nutritional and immune-related biomarkers, both studies underscore the significance of systemic health in cancer prognosis, suggesting that a multifaceted approach to patient assessment could improve prognostic accuracy and treatment strategies.

In another study by Zhang et al. [30], the phenomenon of »Flare« in tumour markers was investigated in advanced gastric cancer patients receiving first-line systemic therapy. Their findings indicated that transient elevations in CEA, CA 19-9, and CA125 levels did not necessarily indicate tumour progression but were associated with poorer overall survival.

In a study by He et al. [31], macrophage migration inhibitory factor (MIF) was identified as a potential prognostic marker in gastric cancer. Their findings demonstrated that elevated MIF expression was associated with poor tumour differentiation, advanced tumour stage, lymph node metastasis, and worse patient survival. Additionally, the knockdown of MIF in gastric cancer cells led to reduced proliferation, suggesting its role as a therapeutic target. Similarly, our study focuses on prognostic biomarkers in oncology, reinforcing the significance of molecular markers in predicting patient outcomes. Both studies highlight the importance of integrating molecular profiling into clinical practice to improve prognostic accuracy and guide targeted therapies.

In Dreher and colleagues' study, CXCR4-directed radioligand therapy (RLT) was shown to have both myeloablative effects and direct antilymphoma activity in heavily pretreated patients with haematological malignancies [32]. Their findings demonstrated a significant decline in leukocyte and platelet levels, along with a transient increase in lactate dehydrogenase (LDH), indicating effective tumour targeting. Importantly, CXCR4-RLT exhibited independent antilymphoma activity before hematopoietic stem cell transplantation (HSCT), suggesting its potential as a therapeutic strategy. Similarly, it emphasises the growing role of targeted therapies in improving treatment outcomes. These findings reinforce the need for integrating molecular-targeted approaches into clinical practice to enhance precision medicine in cancer treatment.

The observed differences in clinical efficacy, immune function, and tumour marker levels between the SOX regimen (oxaliplatin plus S-1) and oxaliplatin monotherapy can be attributed to several potential mechanisms. First, S-1, a fluoropyrimidine-based chemotherapeutic agent, enhances the cytotoxic effects of chemotherapy through its ability to inhibit thymidylate synthase, an enzyme essential for DNA synthesis. This inhibition not only impairs tumour cell proliferation but also potentially strengthens the antitumour effect of oxaliplatin by promoting more effective tumour cell killing. Moreover, S-1's higher oral bioavailability and sustained action compared to capecitabine may lead to more consistent tumour suppression over time, contributing to a higher disease control rate and objective remission rate.

In terms of immune function, the combination of S-1 and oxaliplatin appears to have synergistic effects on immune recovery. Oxaliplatin, a platinum-based chemotherapy, is known to induce immunogenic cell death, releasing tumour antigens and enhancing the presentation of these antigens to immune cells. This process likely stimulates a stronger immune response, promoting the activation and proliferation of T-cells and natural killer (NK) cells. Additionally, S-1 may exert direct immunomodulatory effects by modulating the tumour microenvironment, potentially reducing immune suppression and enhancing overall immune surveillance. This could explain the significant increase in immunoglobulins (IgA, IgG, IgM) and T-cell subsets observed in the SOX group.

Regarding tumour markers, the combination therapy likely works through multiple mechanisms. The reduction in tumour markers such as CA125, CEA, MRP-14, SDF-1, FSP-1, and CXCR4 indicates a more effective inhibition of tumour progression and metastasis. For instance, oxaliplatin-induced DNA damage may promote the release of tumour-associated markers, while the synergistic effects of S-1 could help suppress the activation of pathways involved in metastasis and immune evasion. Additionally, S-1's potential to target cancer-associated fibroblasts and tumour stroma may play a role in reducing stromal-derived factors like SDF-1 and FSP-1, which are involved in metastasis and resistance to chemotherapy.

Together, these mechanisms suggest that the SOX regimen not only enhances tumour cell death but also modulates the immune system and the tumour microenvironment more effectively than oxaliplatin monotherapy, leading to improved clinical outcomes and immune recovery.

Despite the promising findings, this study has several limitations that should be considered when interpreting the results. First, the sample size is relatively small, which may limit the statistical power and generalizability of the findings. A larger, more diverse patient population would provide more robust data and allow for broader conclusions. Second, the retrospective nature of the study introduces potential biases, as treatment selection and patient outcomes may be influenced by factors not accounted for in the analysis. Additionally, the study primarily focused on short-term biomarker changes post-treatment, and long-term follow-up is essential to determine the prognostic value of these biomarkers and their correlation with overall survival and disease recurrence. Future research with a prospective design and more extended follow-up periods is needed to validate these results and better understand the role of serum biomarkers in predicting treatment response and resistance in gastric cancer.

## Conclusion

The SOX regimen significantly improves clinical efficacy, enhances immune function, and reduces tumour marker levels in gastric cancer patients compared to oxaliplatin monotherapy. Serum biomarkers may serve as valuable tools for monitoring treatment response. However, the study's small sample size and short-term follow-up limit its generalizability. More significant, long-term studies are needed to confirm these findings and better understand the prognostic value of these biomarkers in gastric cancer treatment.

## Dodatak

### Acknowledgements

The authors express their gratitude to all the medical staff and researchers who contributed to this study, especially to the patients and their families, for their participation and support.

### Funding

This study received no funding.

### Ethical approval

This study was conducted in accordance with the ethical guidelines, and approval was obtained prior to the initiation of research. Written informed consent was obtained from all participants.

### Data availability

The datasets used and analysed during this study are available from the corresponding authors upon reasonable request.

### Authors' contributions

Ying Huang and Yueming Hu contributed equally to this work and share the first authorship. Yufeng Ni and Fengwei Gu designed and supervised the study. Ying Huang and Yueming Hu performed data collection and analysis. All authors contributed to the manuscript writing and approved the final version.

### Conflict of interest statement

All the authors declare that they have no conflict of interest in this work.

## References

[1] Bray F, Laversanne M, Sung H, Ferlay J, Siegel R L, Soerjomataram I, Jemal A (2024). Global cancer statistics 2022: GLOBOCAN estimates of incidence and mortality worldwide for 36 cancers in 185 countries. CA Cancer J Clin.

[2] Ilić M, Ilić I (2022). Epidemiology of stomach cancer. World J Gastroenterol.

[3] Morgan E, Arnold M, Camargo M, Gini A, Kunzmann A T, Matsuda T, Meheus F, Verhoeven R H A, Vignat J, Laversanne M, Ferlay J, Soerjomataram I (2022). The current and future incidence and mortality of gastric cancer in 185 countries, 2020-40: A population-based modelling study. EClinicalMedicine.

[4] Shirani M, Pakzad R, Haddadi M H, Akrami S, Asadi A, Kazemian H, Moradi M, Kaviar V H, Zomorodi A R, Khoshnood S, Shafieian M, Tavasolian R, Heidary M, Saki M (2023). The global prevalence of gastric cancer in Helicobacter pylori-infected individuals: A systematic review and meta-analysis. BMC Infect Dis.

[5] Hu B, El H N, Sittler S, Lammert N, Barnes R, Meloni-Ehrig A (2012). Gastric cancer: Classification, histology and application of molecular pathology. J Gastrointest Oncol.

[6] Toh J W T, Wilson R B (2020). Pathways of Gastric Carcinogenesis, Helicobacter pylori Virulence and Interactions with Antioxidant Systems, Vitamin C and Phytochemicals. Int J Mol Sci.

[7] Hnatyszyn A, Szalata M, Zielińska A, Wielgus K, Danielewski M, Hnatyszyn P T, Pławski A, Walkowiak J, Słomski R (2024). Mutations in Helicobacter pylori infected patients with chronic gastritis, intestinal type of gastric cancer and familial gastric cancer. Hered Cancer Clin Pract.

[8] Ko K (2024). Risk Factors of Gastric Cancer and Lifestyle Modification for Prevention. J Gastric Cancer.

[9] Baccili Cury Megid T, Farooq A R, Wang X, Elimova E (2023). Gastric Cancer: Molecular Mechanisms, Novel Targets, and Immunotherapies: From Bench to Clinical Therapeutics. Cancers (Basel).

[10] Matsuoka T, Yashiro M (2023). Molecular Insight into Gastric Cancer Invasion-Current Status and Future Directions. Cancers (Basel).

[11] Nevo Y, Ferri L (2023). Current management of gastric adenocarcinoma: A narrative review. J Gastrointest Oncol.

[12] Yeh J H, Yeh Y S, Tsai H L, Huang C W, Chang T K, Su W C, et al (2022). Neoadjuvant Chemoradiotherapy for Locally Advanced Gastric Cancer: Where Are We at?. Cancers (Basel).

[13] Guan W L, He Y, Xu R H (2023). Gastric cancer treatment: Recent progress and future perspectives. J Hematol Oncol.

[14] Bilici A (2014). Treatment options in patients with metastatic gastric cancer: Current status and future perspectives. World J Gastroenterol.

[15] Radford M, Abushukair H, Hentzen S, Cavalcante L, Saeed A (2023). Targeted and Immunotherapy Approaches in HER2-Positive Gastric and Gastroesophageal Junction Adenocarcinoma: A New Era. Journal of Immunotherapy and Precision Oncology.

[16] Charkhchi P, Cybulski C, Gronwald J, Wong F O, Narod S A, Akbari M (2020). CA125 and Ovarian Cancer: A Comprehensive Review. Cancers (Basel).

[17] Desai S, Guddati A K (2023). Carcinoembryonic Antigen, Carbohydrate Antigen 19-9, Cancer Antigen 125, Prostate-Specific Antigen and Other Cancer Markers: A Primer on Commonly Used Cancer Markers. World J Oncol.

[18] Simpson K D, Templeton D J, Cross J V (2012). Macrophage Migration Inhibitory Factor Promotes Tumor Growth and Metastasis by Inducing Myeloid-Derived Suppressor Cells in the Tumor Microenvironment. J Immunol.

[19] Mora Barthelmess R, Stijlemans B, van Ginderachter J A (2023). Hallmarks of Cancer Affected by the MIF Cytokine Family. Cancers (Basel).

[20] Grieb G, Merk M, Bernhagen J, Bucala R (2010). Macrophage migration inhibitory factor (MIF): A promising biomarker. Drug News Perspect.

[21] Hussain S, Peng B, Cherian M, Song J W, Ahirwar D K, Ganju R K (2020). The Roles of Stroma-Derived Chemokine in Different Stages of Cancer Metastases. Front Immunol.

[22] Wright K, Ly T, Kriet M, Czirok A, Thomas S M (2023). Cancer-Associated Fibroblasts: Master Tumor Microenvironment Modifiers. Cancers (Basel).

[23] Chatterjee S, Behnam Azad B, Nimmagadda S (2014). The intricate role of CXCR4 in cancer. Adv Cancer Res.

[24] Iwanicki-Caron I, di Fiore F, Roque I, Astruc E, Stetiu M, Duclos A, Tougeron D, Saillard S, Thureau S, Benichou J, Paillot B, Basuyau J P, Michel P (2008). Usefulness of the Serum Carcinoembryonic Antigen Kinetic for Chemotherapy Monitoring in Patients With Unresectable Metastasis of Colorectal Cancer. J Clin Oncol.

[25] Kang S, Kim T, Seo S, Kim B, Bae D, Park S (2011). Interaction between preoperative CA-125 level and survival benefit of neoadjuvant chemotherapy in advanced epithelial ovarian cancer. Gynecol Oncol.

[26] Wang Y, Huang X, Mo M, Li J, Jia X, Shao Z, Shen Z, Wu J, Liu G (2015). Serum Tumor Marker Levels might have Little Significance in Evaluating Neoadjuvant Treatment Response in Locally Advanced Breast Cancer. Asian Pac J Cancer Prev.

[27] Yamao T, Kai S, Kazami A, Koizumi K, Handa T, Takemoto N, Maruyama M (1999). Tumor Markers CEA, CA19-9 and CA125 in Monitoring of Response to Systemic Chemotherapy in Patients with Advanced Gastric Cancer. Jpn J Clin Oncol.

[28] Sun Z, Zhang N (2014). Clinical evaluation of CEA, CA19-9, CA72-4 and CA125 in gastric cancer patients with neoadjuvant chemotherapy. World J Surg Oncol.

[29] Du Z, Sun H, Zhao R, Deng G, Pan H, Zuo Y, Huang R, Xue Y, Song H (2023). Combined with prognostic nutritional index and IgM for predicting the clinical outcomes of gastric cancer patients who received surgery. Front Oncol.

[30] Zhang F, Zhai M, Yang J, Zhao L, Lin Z, Wang J, et al (2022). 'FLARE' of tumor marker in advanced gastric cancer treated with first-line systemic therapy. Therap Adv Gastroenterol.

[31] He L (2015). Macrophage migration inhibitory factor as a potential prognostic factor in gastric cancer. World J Gastroenterol.

[32] Dreher N, Dörrler A, Kraus S, Higuchi T, Serfling S E, Samnick S, Einsele H, Grigoleit G U, Buck A K, Werner R A (2024). C-X-C Motif Chemokine Receptor 4-Targeted Radioligand Therapy in Hematological Malignancies-Myeloablative Effects, Antilymphoma Activity, and Safety Profile. Clin Nucl Med.

